# Investigation into relationships between design parameters and mechanical properties of 3D printed PCL/nHAp bone scaffolds

**DOI:** 10.1371/journal.pone.0288531

**Published:** 2023-07-14

**Authors:** Zahra Yazdanpanah, Nitin Kumar Sharma, Amanda Zimmerling, David M. L. Cooper, James D. Johnston, Xiongbiao Chen

**Affiliations:** 1 Division of Biomedical Engineering, College of Engineering, University of Saskatchewan, Saskatoon, Saskatchewan, Canada; 2 Department of Mechanical Engineering, College of Engineering, University of Saskatchewan, Saskatoon, Saskatchewan, Canada; 3 Department of Anatomy Physiology and Pharmacology, College of Medicine, University of Saskatchewan, Saskatoon, Saskatchewan, Canada; University of Southampton, UNITED KINGDOM

## Abstract

**Background:**

Scaffolds are of great importance in tissue engineering applications as they provide a mechanically supportive environment for cellular activity, which is particularly necessary for hard tissues such as bone. Notably, the mechanical properties of a scaffold vary with differing design parameters such as those related to scaffold height and internal structure. Thus, the present study aimed to explore the relationship between design parameters and mechanical properties of composite polycaprolactone (PCL) and nano-hydroxyapatite (nHAp) scaffolds fabricated by three-dimensional (3D) printing.

**Methods:**

We designed and printed scaffolds with different internal structures (lattice and staggered) and varying heights (4, 6, 8 and 10 layers), and consistent porosity (50%) for the purpose of comparison. Then, we examined the scaffold microstructure (pore size and penetration between layers) using scanning electron microscopy (SEM) and mechanical properties (elastic modulus and yield strength) using compressive testing.

**Results:**

Our results illustrated that the microstructural parameters were related to scaffold design. At higher heights, pore size increased while penetration between layers decreased; thus, mechanical properties were affected. Results of mechanical testing demonstrated that for lattice scaffolds, elastic modulus was similar for 6 vs 4, and 8 vs 4 layers but ~33% lower for 10 layers vs 4 layers. Similarly, yield strength was comparable for 6 vs 4, and 8 vs 4 layers but ~27% lower for 10 layers vs 4 layers. With staggered scaffolds, when compared to 4-layer results, elastic modulus was similar for 6 layers but was ~43% lower for 8 layers and ~38% lower for 10 layers. Staggered scaffolds had ~38%, ~51%, and ~76% lower yield strength when the number of layers were increased from 4 to 6, 8, and 10 layers, respectively. When comparing lattice and staggered scaffolds with the same layer number, elastic modulus was similar, apart from 8-layer scaffolds where the staggered design was ~42% lower than lattice. Yield strength was similar between 4-layer staggered and lattice scaffolds, while staggered scaffolds with 6, 8, and 10 number of layers showed ~43%, ~45%, ~68% lower strength, respectively, than those found in lattice scaffolds with the same layer numbers.

**Conclusions:**

Mechanical properties of 3D printed scaffolds depended on scaffold height for both lattice and staggered internal structures. Staggered scaffolds had lower mechanical properties than the lattice scaffolds with the same height and were more sensitive to the change in scaffold height. Taken together, lattice scaffolds demonstrated the advantages of more stable mechanical properties over staggered scaffolds. Also, scaffolds with lower height were more promising in terms of mechanical properties compared to scaffolds with greater height.

## 1 Background

In recent decades, scaffold-based bone tissue engineering (BTE) has emerged as an effective solution to overcome the limitations and obstacles that exist for conventional bone grafting approaches (*e*.*g*., autografts, allografts) [1–4]. In BTE, a scaffold provides a temporary template for mechanical support, allowing the construct to withstand external forces throughout the process of tissue regeneration [3–6].

Three-dimensional (3D) printed scaffolds composed of bioactive ceramic nano-hydroxyapatite (nHAp) and polymer polycaprolactone (PCL) have gained considerable interest in BTE [7–9]. The ceramic phase of nHAp provides bioactivity and high compressive modulus while the polymeric phase of PCL is easily processed via 3D printing techniques and provides biodegradability, toughness, and flexibility [10–15]. Despite the substantial number of investigations into extrusion-based 3D printed PCL/nHAp scaffolds, reported values of elastic modulus are often in conflict. For example, PCL/40% (wt./v) HAp scaffolds with 10-mm height investigated by Dorj *et al*. [16] have shown higher elastic modulus (*E*≈40 MPa) than that found in PCL/40% (wt.) HAp scaffolds (*E*≈10 MPa) with 4-mm height, as studied by Li *et al*. [17]. Similarly, different elastic moduli have been reported in the literature for PCL/20% (wt.) HAp scaffolds. For example, an elastic modulus of ~76 MPa was reported by Huang *et al*. [13] for PCL/20% (wt.) HAp scaffolds with 5.0 mm height [13] while Kim *et al*. [18] found an elastic modulus of ~61 MPa for PCL/20% (wt.) HAp scaffolds with 2.4 mm height [18]. Additionally, different elastic moduli have been reported for PCL/10% (wt.) HAp scaffolds investigated with different heights (*E*≈60 MPa for 5-mm scaffolds by Huang *et al*. [13] versus *E*≈44 MPa for 2.4-mm scaffolds by Kim *et al*. [18]). This indicates that scaffold height could be an influential factor on the mechanical properties of 3D printed scaffolds and may explain these inconsistent and/or conflicting reports.

In addition to height, mechanical properties of 3D printed porous scaffolds have also been found to be affected by many other design-related parameters [19–25]. Among these parameters, the internal structure of a scaffold, referring to the arrangement of strands, has been found to affect the elastic modulus of scaffolds upon compression [1,15]. For instance, PCL scaffolds [26] and PCL/nHAp scaffolds [27] with misaligned strands between subsequent layers exhibited lower elastic modulus compared to scaffolds with aligned strands. Although scaffolds with aligned strands seem to provide a greater supportive structure mechanically, those with misaligned strands have been found to be more promising for cellular activities (*e*.*g*., cell-seeding efficacy) [28]. This is due to the higher number of potential contact points between cells and strands within misaligned scaffolds [28].

A crucial, but often overlooked, scaffold-design parameter in the BTE literature, is the number of printed layers (leading to different heights). Thus, in the present study, we aimed to investigate whether the mechanical properties of PCL/30% (wt.) nHAp scaffolds are affected by scaffold height. To this end, we studied the effect of height for scaffolds with and without aligned strands, hereafter referred to as lattice and staggered scaffolds, respectively. The heights of the scaffolds were determined by the number of 3D printed layers (4, 6, 8, and 10 layers). Microstructural features of the 3D printed scaffolds (pore size and layer penetration) were also evaluated to gain a better understanding of the relationships between scaffold height and mechanical properties.

## 2 Methods

### 2.1 Scaffold materials

PCL pellets (Mn = 45,000, Product No. 704205-100G) and nHAp powder (particle size < 200 nm, Product No. 677418-25G) were purchased from Sigma-Aldrich Canada Co.

### 2.2 Preparation of composite scaffold material

A solvent-free melt blending technique was used to prepare the composite material, consisting of PCL and nHAp with the weight ratio of 70% (wt.) to 30% (wt.), respectively. As per our previous study [14], PCL pellets were first melted in a beaker followed by the slow addition of nHAp powder under constant stirring to make a homogenous mixture. The resulting slurry was then left to solidify and cut into small pieces for scaffold fabrication using 3D printing.

### 2.3 Design and 3D printing of composite scaffolds

**Fig 1:**
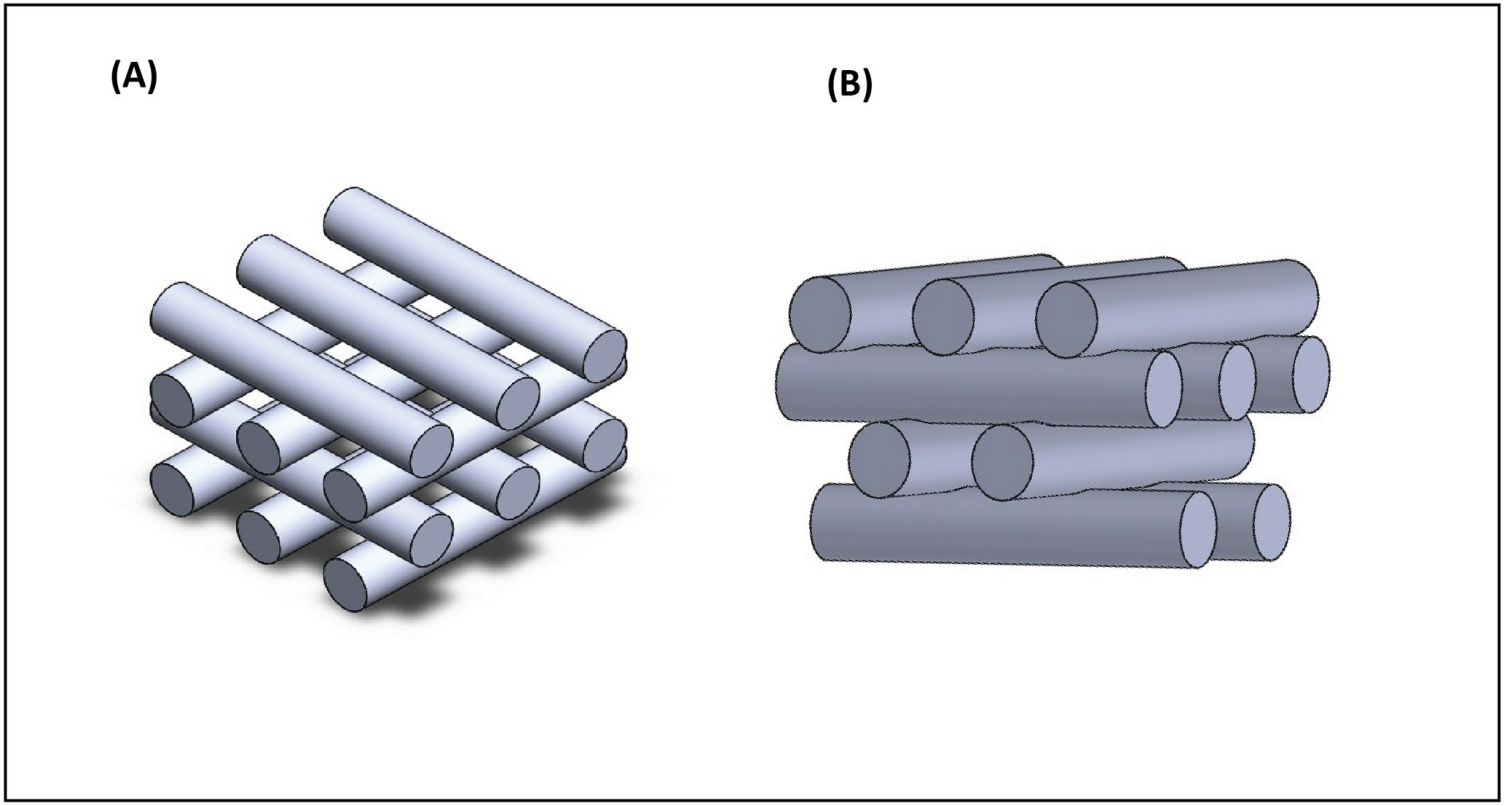
Schematic illustrations of different scaffold internal structures; A) lattice, B) staggered.

Square scaffolds (10 mm × 10 mm) were designed (Magics 13 EnvisionTEC software) for 3D printing. A needle with an internal diameter of 0.510 mm was used and the layer thickness was set at 80% of the strand diameter (*i*.*e*., 0.408 mm) to account for gravitational spreading [14,29]. A distance between strands (*L*) of 1 mm was used; this distance was calculated from the center of strands. Further, lattice and staggered scaffolds with a 0°/90° lay-down pattern using no interlayer offset and 50% interlayer offset were designed, respectively (Fig 1A and 1B). In the lattice scaffold (Fig 1A), strands were located on top of each other from the bottom to the top of scaffold. In the staggered scaffold (Fig 1B), the first and second layers were on top of each other, then the subsequent third and fourth layers were shifted with an interlayer offset value equal to half the distance between strands in both the 0° and 90° directions.

PCL/30% (wt.) nHAp scaffolds were 3D printed using an extrusion-based 3D Bioplotter Manufacturer Series system (EnvisionTEC GmbH) equipped with a high temperature printing head. Lattice and staggered scaffolds with 4, 6, 8, and 10 layers were 3D printed (n = 3 for each structure and each layer count); thus, total number of 24 samples were 3D printed.

**Fig 2:**
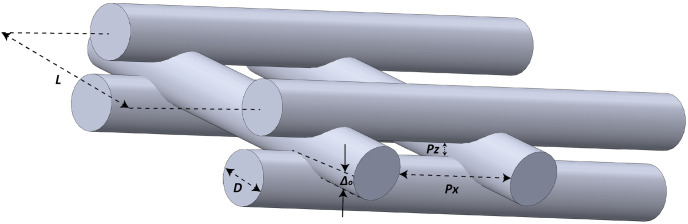
Schematic illustration of an actual 3D printed scaffold made by extrusion-based 3D printer; *D* = strand diameter, *L* = distance between strands, *Δ*_*0*_ = layer penetration, *Px* = pore width, *Pz* = pore height.

A schematic illustration of an actual 3D printed scaffold is displayed in Fig 2, showing the penetration between subsequent layers, which differs from the ideal design (Fig 1A). The difference between a designed scaffold and an actual 3D printed scaffold is known as printability. Printability can be characterized by various parameters such as penetration between layers, and/or differences in pore size.

Scaffolds with total porosity of 50% were 3D printed. Apparent porosity of the scaffolds was measured by calculating the scaffold apparent density *(i*.*e*., scaffold mass divided by volume of the scaffold) and the strand density using Eqs 1 and 2, respectively:

$$Apparent\;porosity\;\left(\%\right)=\left(1-\frac{\rho_{app.}}{\rho_{s}}\right)\times 100$$ (1)

where *ρ*_*app*_. and *ρ*_*s*_ are the apparent density of scaffold and the strand density, respectively. Strand density was also measured using Eq 2:

$$\;\;\rho_{s}=X_{PCL}\rho_{PCL}+X_{nHAp}\rho_{nHAp}$$ (2)

where the *X*_*PCL*_ and *X*_*nHAp*_ are the weight fraction of PCL (70%) and nHAp (30%), respectively, while *ρ*_*PCL*_ and *ρ*_*nHAp*_ refer to the density of PCL (1.145 g/cm^3^) and nHAp (3.14 g/cm^3^), respectively [15].

### 2.4 Microstructural study

The microstructure of the 3D printed scaffolds with differing numbers of layers were evaluated using scanning electron microscopy (SEM) (Hitachi SU8010). A total of 8 scaffolds were selected for SEM analysis: one per studied structure (*i*.*e*., lattice and staggered) and layer number (*i*.*e*., 4–10 layers) group; SEM images from cross-sections were taken. Each scaffold was coated with 10 nm of gold (Quorum Q150TES Sputter Coater) and then scanned under high vacuum at an accelerating voltage of 3.0 kV. Of note, for the 10-layer scaffolds, visualizing all the layers within a single image was not feasible due to limitations associated with the lowest SEM magnification. SEM images were used to measure microstructural parameters including the amount of penetration between layers (*Δ*_*0*_) as well as pore width (*Px*) and pore height (*Pz*) (or layer gap), which are displayed in the illustration in Fig 2. Of note, penetration between layers pertains to the amount of diffusion between strands in subsequent layers. All strand diameters and distances between strands, as shown in Fig 2, were also measured. ImageJ software (National Institutes of Health [30]) was used to measure the microstructural parameters. Measurements pertain to only one lay-down direction since SEM images were taken from one side only.

### 2.5 Mechanical testing

**Fig 3:**
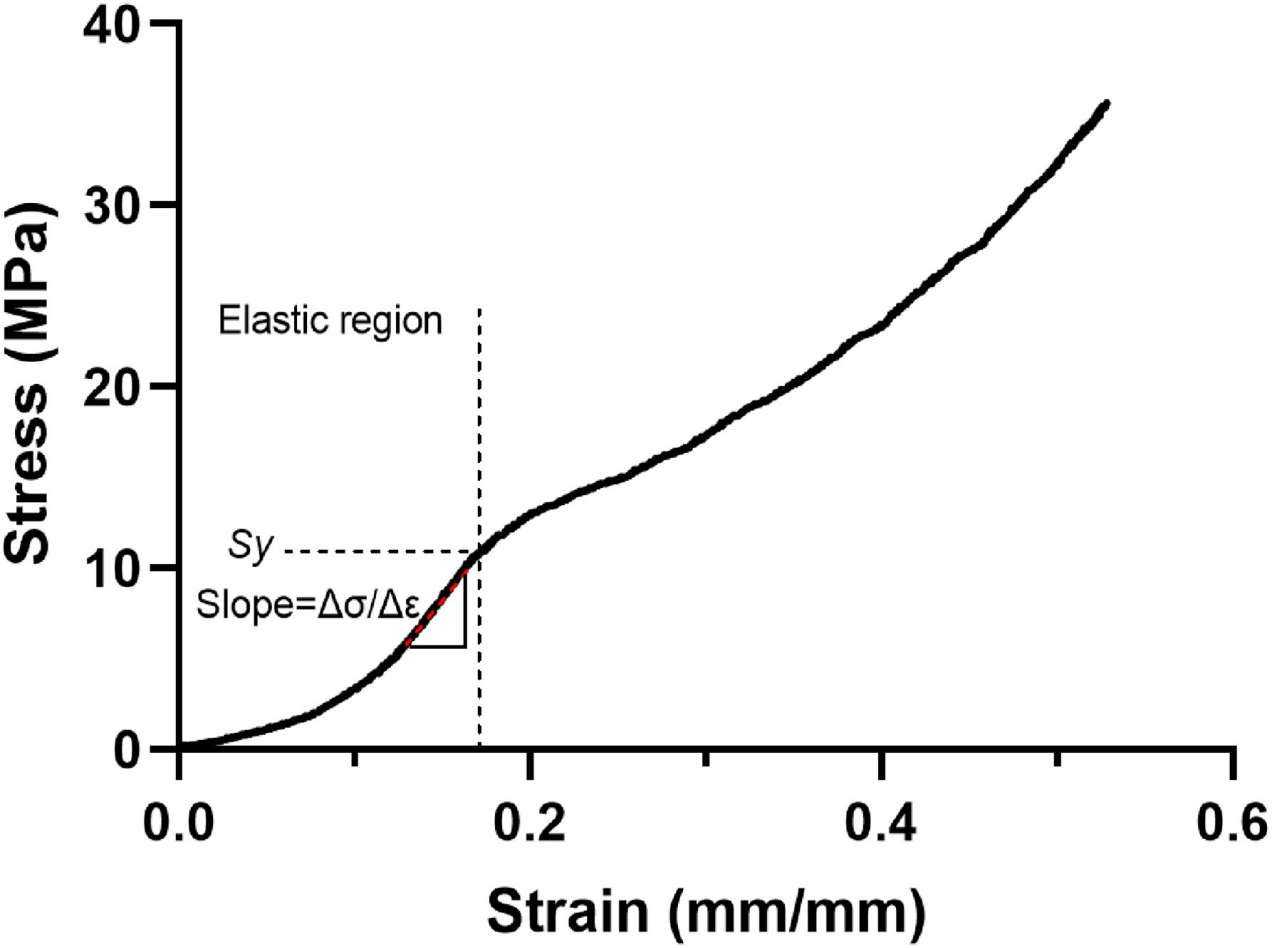
A representative stress-strain curve of a 3D printed PCL/30% (wt.) nHAp scaffold obtained after compressive testing displaying the linear region to derive E ($\text{slope}=\frac{\Delta \sigma }{\Delta \varepsilon }$) and the point pertaining to Sy.

The scaffolds were mechanically tested in compression (MTS Bionix^®^ Servohydraulic Test System) with a 5 kN load cell and a crosshead speed of 1.0 mm/min. For each structure and number of layers, three scaffolds were tested. They were compressed to 75% of their total initial height. Apparent elastic modulus (*E*) was derived from the slope of the linear region (red dashed line) of stress-strain curve in the elastic region using linear regression. Yield strength (*Sy*) was defined as the point where the stress-strain curve diverged from the linear region (Fig 3).

### 2.6 Statistical analysis

Elastic moduli and yield strengths of lattice and staggered scaffolds were assessed as a function of number of printed layers. ANOVA was used to compare the mechanical properties of different groups with respect to scaffolds with 4 layers. Additionally, lattice and staggered scaffolds with the same layer number were compared in terms of mechanical properties to assess the influence of internal structure. If an overall statistical difference was observed, Bonferroni correction was used to perform *post-hoc* pairwise comparisons. In all analyses, *p* ≤ 0.05 was considered the threshold of statistical significance. The data are presented as mean (M) ± standard deviation (SD). Statistical analyses were performed using GraphPad Prism 9.4.1.

## 3 Results

### 3.1 Microstructural study

**Fig 4:**
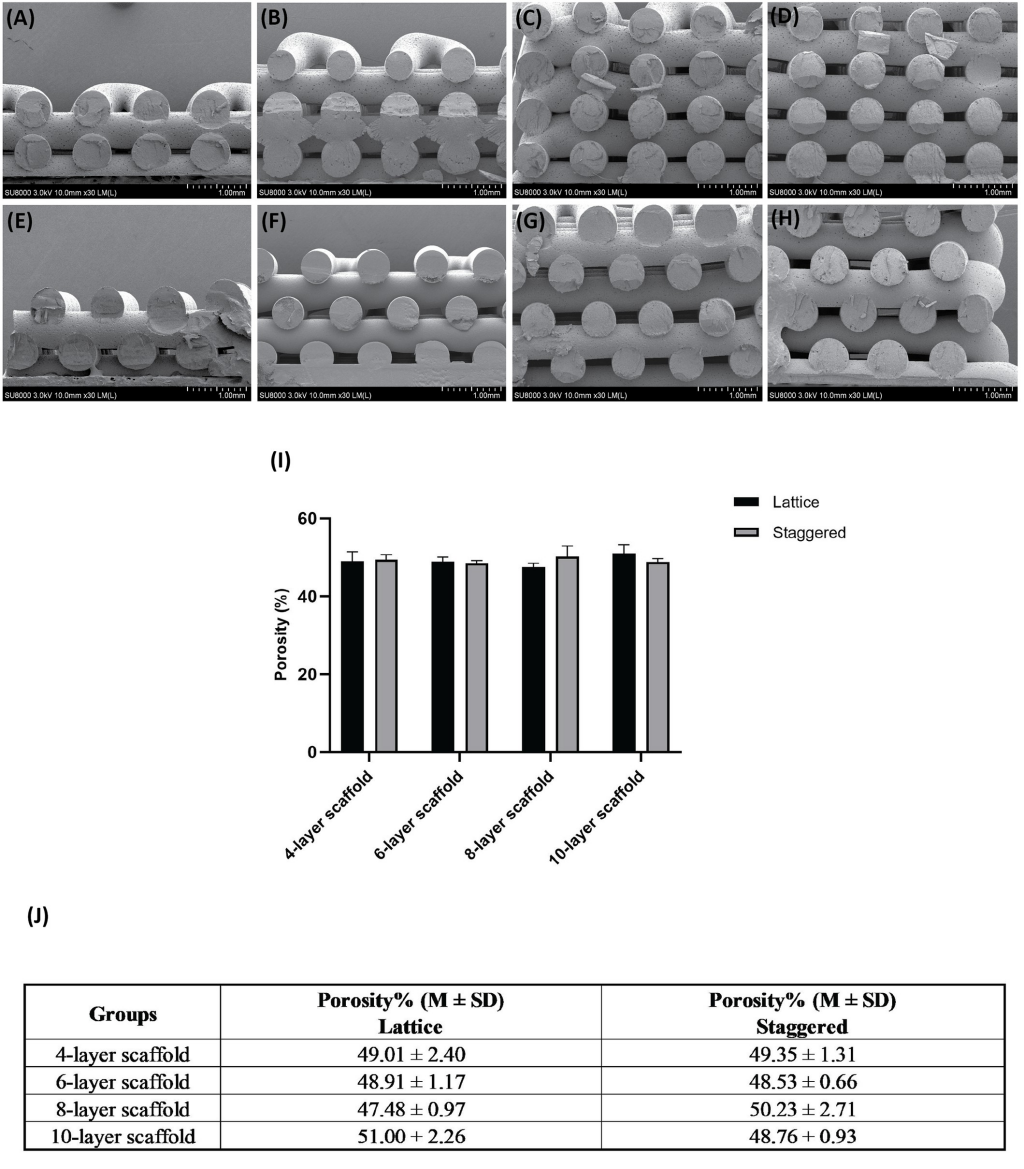
SEM images of lattice scaffolds; A) 4 layers, B) 6 layers, C) 8 layers, D) 10 layers, and staggered scaffolds; E) 4 layers, F) 6 layers, G) 8 layers, H) 10 layers. I) Bar graphs showing apparent porosities of lattice and staggered scaffolds with different numbers of layers (values pertain to the total porosity of scaffolds and not the porosity of each 3D printed layer), J) Quantitative values (M ± SD) of porosities for different scaffold groups.

SEM images showed highly porous structures with interconnected pores from the top to the bottom of each scaffold (Fig 4A–4H). Apparent porosity (~ 50%) for lattice as well as staggered scaffolds with differing numbers of layers is plotted in Fig 4I and quantitative values (M ± SD) of measured porosities for scaffolds are listed in Fig 4J.

Quantitative values (M ± SD) pertaining to the diameter of strands and distance between strands for lattice and staggered scaffolds are given in Table 1 and statistical comparisons are given in S1 and S2 Tables in S1 File. No statistical differences were found for distance between strands in the different scaffold groups. Regarding strand diameter, 4-layer staggered scaffolds showed a higher (*p ≤* 0.05) diameter than that of found in 6-layer staggered scaffolds.

**Table 1:** Strand diameter and distance between strands for lattice and staggered scaffolds (Data are shown as M ± SD).

Number of layers	*D* (mm)-Lattice	*D* (mm)-Staggered	*L* (mm)-Lattice	*L* (mm)-Staggered
4 layers	0.617 ± 0.024	0.619 ± 0.039	0.988 ± 0.016	0.970 ± 0.032
6 layers	0.589 ± 0.087	0.536 ± 0.022	0.955 ± 0.042	0.947 ± 0.014
8 layers	0.584 ± 0.028	0.589 ± 0.049	0.955 ± 0.033	0.976 ± 0.029
10 layers	0.589 ± 0.038	0.580 ± 0.068	0.984 ± 0.033	0.970 ± 0.036

**Fig 5:**
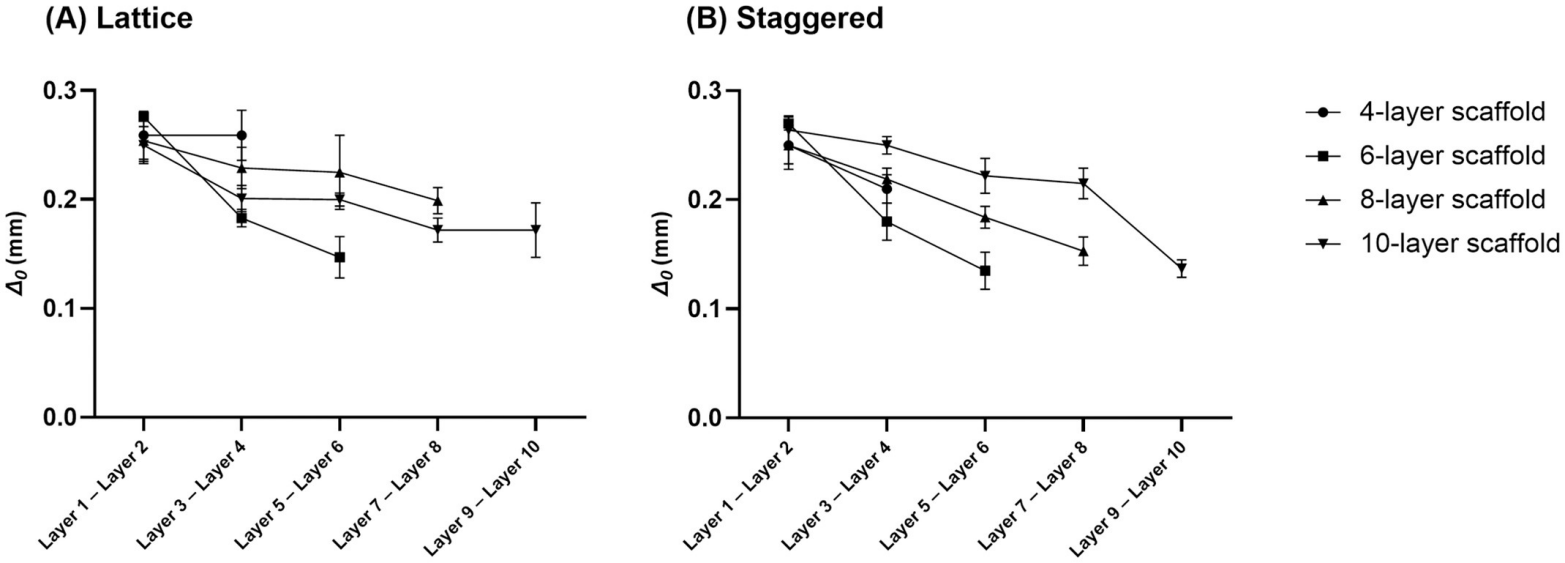
Amount of penetration between subsequent printed layers; A) lattice scaffolds, B) staggered scaffolds (Data are presented as M ± SD).

**Fig 6:**
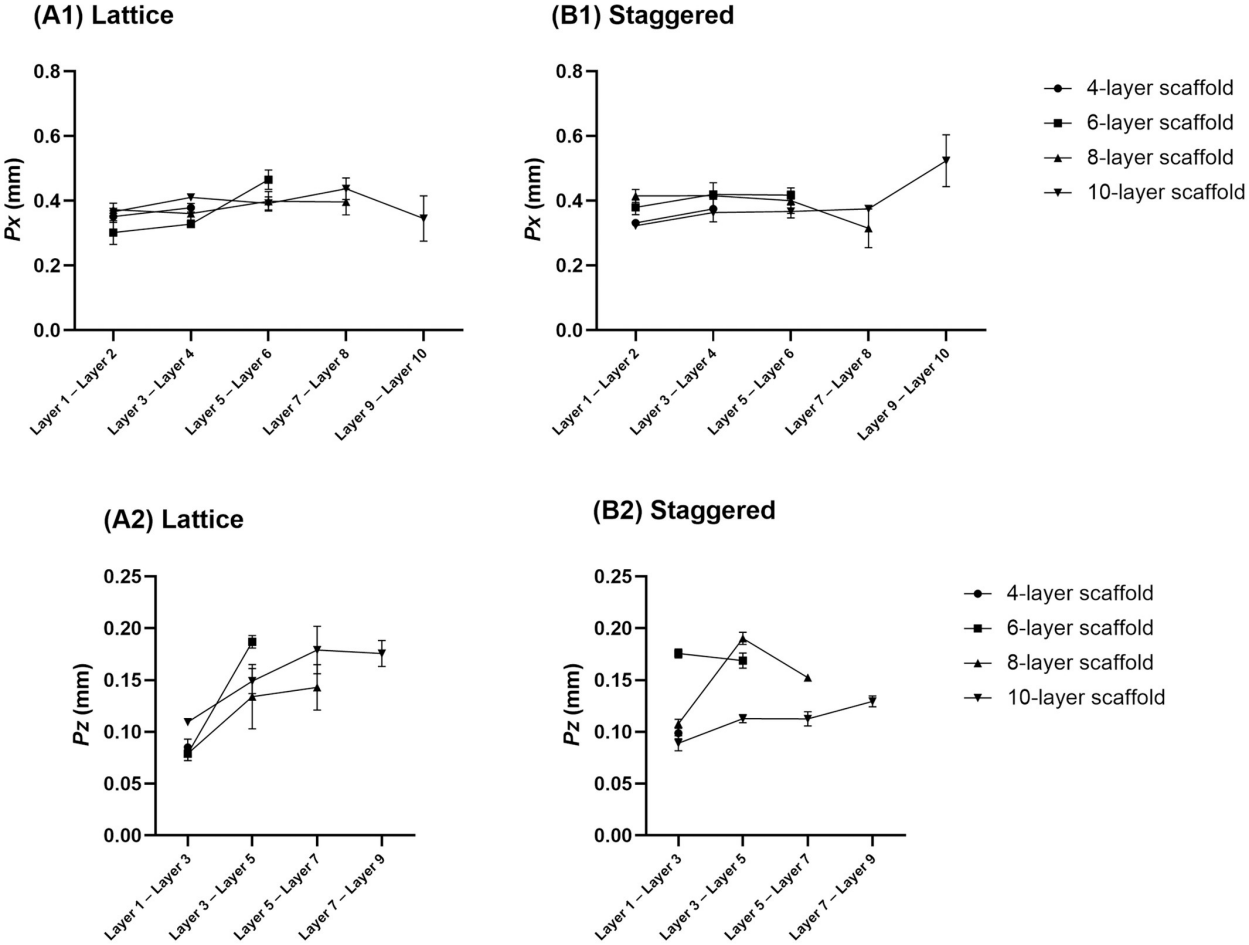
Size of pore width (Px) and pore height (Pz) for scaffolds with different numbers of layers; A1-A2) lattice scaffold, B1-B2) staggered scaffold (Data are presented as M ± SD).

From the printability point of view, no penetration between layers is considered in a designed model (Fig 1A and 1B); however, actual 3D printed scaffolds revealed penetration between subsequent layers as depicted by SEM images (Fig 4A–4H). Printability-related measurements are presented in Figs 5 and 6, respectively. Quantitative values (M ± SD) of these parameters (*Δ*_*0*_, *Px*, *Pz*) are listed in S3-S8 Tables in S1 File. Although penetration between subsequent layers followed a general trend of decreasing, the size of pores (*Px* and *Pz*) in lattice and staggered scaffolds demonstrated a trend of increasing along with layer number; however, a few unexpected drops in pore size values by increasing the number of layers were observed. Variation in layer penetration and pore size from the bottom to the top of the scaffolds indicated the importance of assessing each layer in terms of these parameters.

### 3.2 Mechanical testing

**Fig 7:**
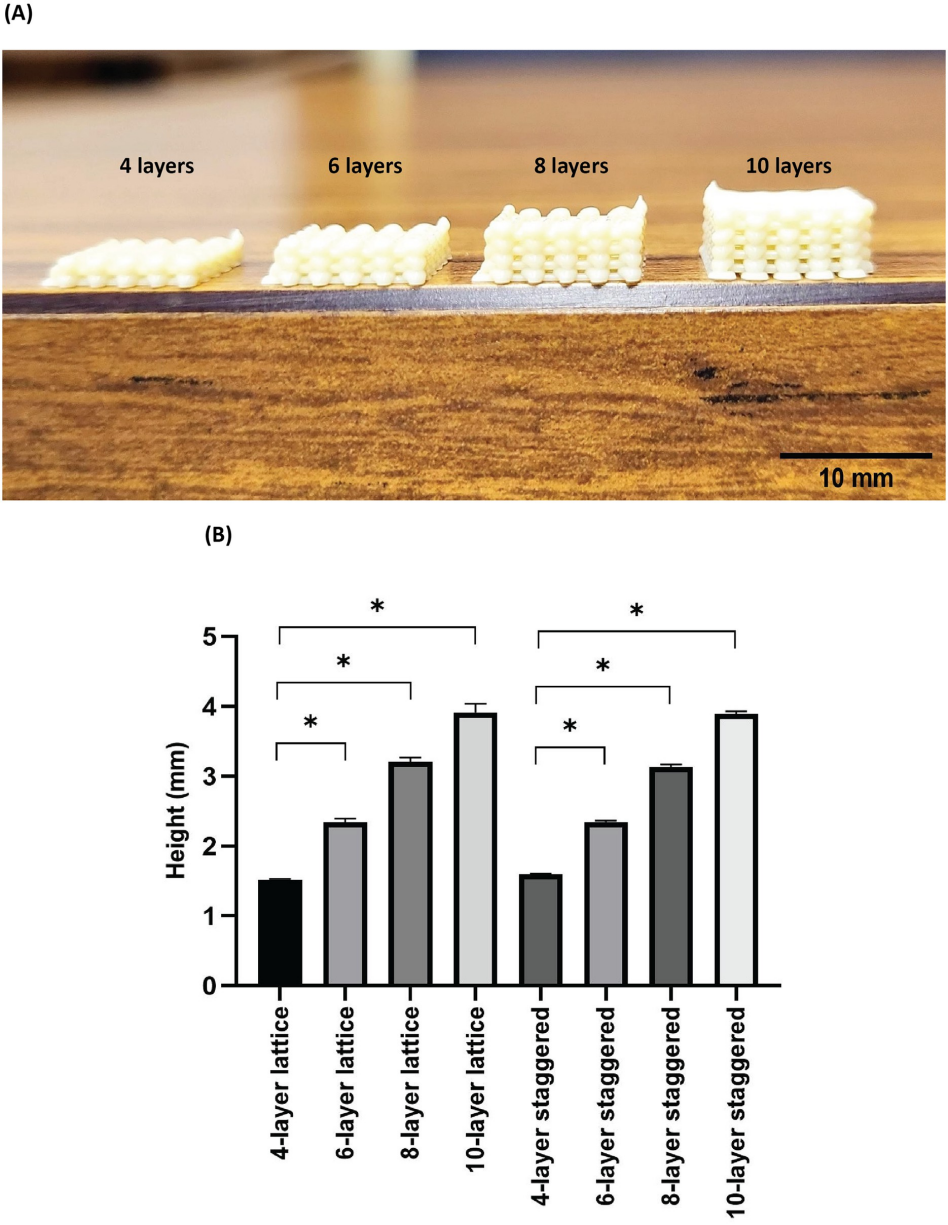
A) Representative images of 3D printed scaffolds with different numbers of layers (4, 6, 8, and 10 layers), B) Bar graphs pertaining to the heights of scaffolds with different numbers of layers (Data are presented as M ± SD, *p ≤ 0.05).

Representative images of scaffolds with different numbers of layers are given in Fig 7A. Heights of scaffolds were measured prior to mechanical testing. Average heights as well as corresponding standard deviations of lattice scaffolds were M = 1.52 mm, SD = 0.01 mm for 4 layers; M = 2.34 mm, SD = 0.06 mm for 6 layers; M = 3.21 mm, SD = 0.06 mm for 8 layers; and M = 3.91 mm, SD = 0.12 mm for 10 layers (Fig 7B). Average heights as well as corresponding standard deviations of staggered scaffolds were M = 1.60 mm, SD = 0.01 mm for 4 layers; M = 2.34 mm, SD = 0.02 mm for 6 layers; M = 3.13 mm, SD = 0.04 mm for 8 layers; and M = 3.90 mm, SD = 0.04 mm for 10 layers (Fig 7B). No statistical differences were found between heights of scaffolds with the same number of layers (*e*.*g*., 4-layer lattice vs 4-layer staggered). Whereas, scaffolds with the same internal structure but varied layer number were different in terms of height (S9 Table in S1 File) (*p ≤* 0.05).

**Fig 8:**
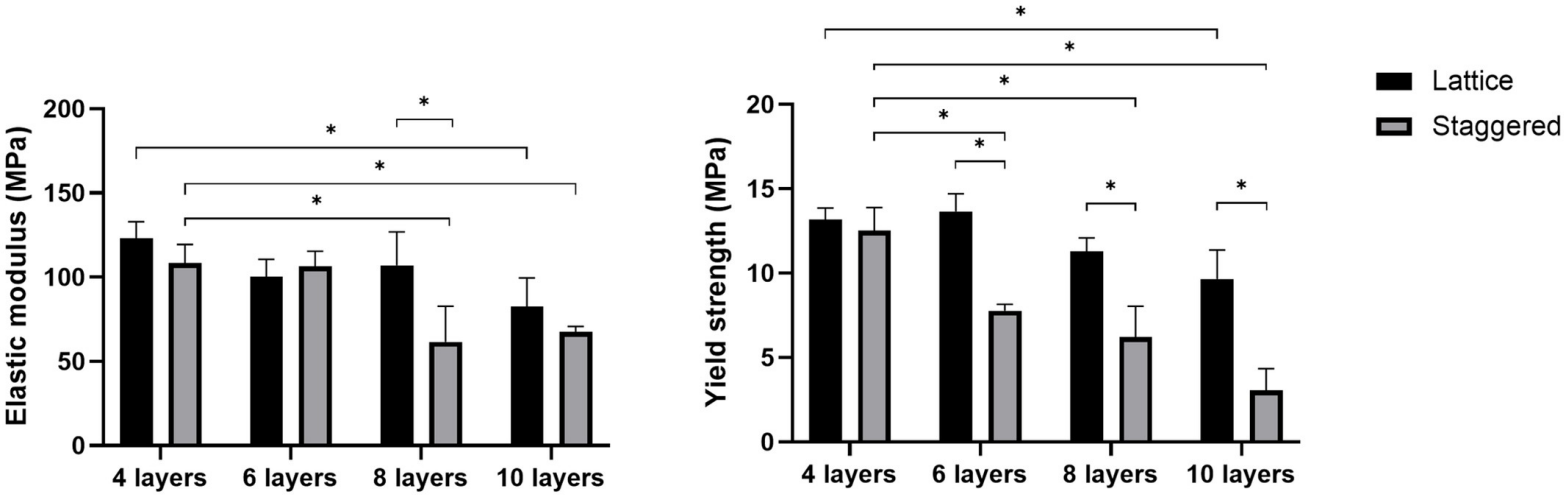
Elastic modulus and yield strength of lattice and staggered scaffolds vs. the number of 3D printed layers (Data are presented as M ± SD, *p ≤ 0.05).

Mechanical properties of lattice and staggered scaffolds with four different layer counts are presented using bar plots shown in Fig 8, and the corresponding quantitative values (M ± SD) are given in Tables 2 and 3.

**Table 2:** Elastic modulus for lattice and staggered scaffolds with differing numbers of layers (Data are presented as M ± SD).

Number of layers	*E* (MPa)-Lattice	*E* (MPa)-Staggered
4 layers	123.07 ± 9.92	108.18 ± 11.31
6 layers	100.31 ± 10.22	106.49 ± 8.87
8 layers	107.13 ± 19.75	61.63 ± 21.20
10 layers	82.43 ± 17.08	67.53 ± 3.22

**Table 3:** Yield strength for lattice and staggered scaffolds with differing numbers of layers (Data are presented as M ± SD).

Number of layers	*Sy* (MPa)-Lattice	*Sy* (MPa)-Staggered
4 layers	13.17 ± 0.69	12.53 ± 1.36
6 layers	13.65 ± 1.06	7.78 ± 0.38
8 layers	11.29 ± 0.81	6.20 ± 1.83
10 layers	9.67 ± 1.71	3.05 ± 1.29

Quantitatively, lattice and staggered scaffolds followed a general trend of lower mechanical properties as the layer number increased (Fig 8).

According to the mechanical testing results (Tables 2 and 3), 10-layer lattice scaffolds exhibited ~33% lower elastic modulus (*p ≤* 0.05) and ~27% lower yield strength (*p ≤* 0.05) compared to that of found in 4-layer lattice scaffolds. Similar elastic moduli and yield strengths were noted for 4 vs 6, and 4 vs 8 layers (*p* > 0.05). For staggered scaffolds, relative to 4-layer scaffolds, 10-layer scaffolds exhibited ~38% lower elastic modulus (*p ≤* 0.05) while 8-layer exhibited ~43% lower elastic moduli (Tables 2 and 3). Similar elastic moduli were noted for 4 vs 6 layers (*p* > 0.05). Staggered scaffolds though showed ~38%, ~51%, and ~76% lower yield strength (*p ≤* 0.05) when the number of layers changed from 4 to 6 layers, 4 to 8 layers, and 4 to 10 layers, respectively. Statistical details are given in S10 and S11 Tables in S1 File.

Comparing lattice and staggered scaffolds with the same layer number (Fig 8, Tables 2 and 3), elastic moduli were similar (*p* > 0.05), apart from 8-layer staggered scaffolds which showed ~42% lower values (*p ≤* 0.05) compared to 8-layer lattice scaffolds (S12 Table in S1 File). Yield strength was similar between 4-layer staggered and lattice scaffolds (*p* > 0.05), while staggered scaffolds with 6, 8, and 10 number of layers showed ~43%, ~45%, ~ 68% lower strength (*p ≤* 0.05), respectively, than those found in lattice scaffolds with the same layer number (S13 Table in S1 File).

**Fig 9:**
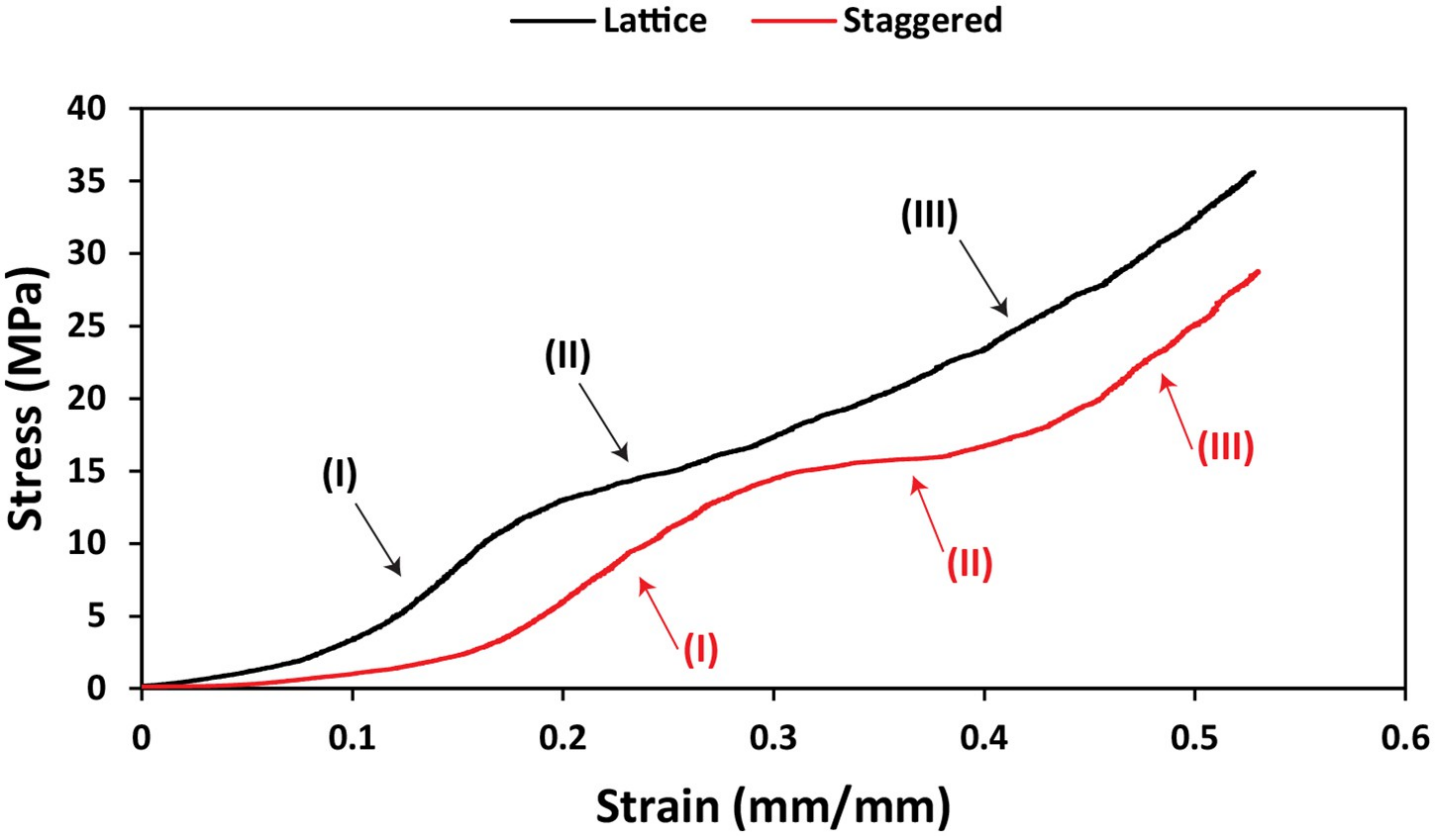
Representative stress-strain curves for lattice and staggered scaffolds with 4 layers upon compression. All three different regions are marked for each internal structure.

Interestingly, representative stress-strain curves obtained from compression tests demonstrated that both lattice and staggered scaffolds yielded three regions regardless of layer number. In other words, both internal structures showed an elastic region (I) followed by a plateau region (II) and eventually a third region (III) in which a steep increase in stress occurred with increasing the compressive strain (Fig 9).

## 4 Discussion

The findings of this research indicated that mechanical properties of 3D porous PCL/30% (wt.) nHAp scaffolds are affected by scaffold height. For lattice scaffolds (Fig 8), elastic modulus and yield strength were found to be similar up to eight layers. Although staggered scaffolds (Fig 8) exhibited consistent elastic modulus up to six layers, their strength dropped by increasing the number of layers more than four.

Microstructural evaluation indicated that parameters related to printability could explain different mechanical properties for scaffolds with different heights. Penetration between layers, which has an influence on the bonding between layers, is an important printability factor [31,32]. When a fresh strand is laid on a dried strand, the contact region spreads resulting in penetration [32]. With an increasing number of layers, strands lower in the scaffold experience higher weight leading to higher penetration (Fig 5) and bonding between layers compared to strands at the top. As a result, there is a higher chance of layer displacement with increased height due to comparatively less layer bonding occurring at higher heights compared to lower heights; hence, strength and stiffness are reduced. Increased *Pz* at higher heights (Fig 6), which shows a greater gap between subsequent layers, also confirms the reduced amount of bonding at higher heights for lattice and staggered scaffolds. Additionally, reduced elastic modulus and strength in scaffolds with higher heights could be attributed to slightly bigger pore width (*Px*) (Fig 6).

Previous research has reported the relationship between mechanical properties and scaffold height, where scaffolds with varying numbers of layers were fabricated. Elastic modulus of 3D printed alginate hydrogel scaffolds has been found to increase with increasing number of 3D printed layers [29]. In addition, 3D printed poly(lactic acid) [33] scaffolds with more layers showed higher elastic modulus compared to scaffolds with fewer layers, while the yield strength was not affected by layer count. Although these reports are not in agreement with our findings regarding PCL/30% (wt.) nHAp scaffolds, they indicate that height should be considered when designing a 3D printed scaffold for tissue engineering applications. A potential reason for this discrepancy between our findings and previous research [29,33] could be attributed to the differences in the scaffold material, which subsequently could influence the amount of penetration between layers and/or pore size as the layer number changes.

Ideally, a bone scaffold should not only demonstrate mechanical properties close to those of natural bone, it should also have structural features (*e*.*g*., controlled pore size, porosity) to help facilitate cellular activity [34]. As a general guideline, it has been reported that scaffolds with pore size larger than 0.300 mm are appropriate for BTE [35] and in a prediction-based model [36], scaffolds with 50% porosity have been shown to improve vascularization. Also, experimental findings via *in-vitro* and *in-vivo* examinations have shown that PCL/nHAp scaffolds with the total porosity of ~ 50% were promising in terms of osteogenic activity [37]. The scaffolds investigated in our study met these structural requirements in terms of porosity level and pore size (Fig 6A1 and 6B1), although *in-vitro* biological studies were not carried out as a part of this research. Our scaffolds also had elastic moduli and yield strengths in the range of 62–123 MPa and 3–14 MPa, respectively, which matches the mechanical properties of human trabecular bone (*E* ~ 14–121 MPa [38], *Sy* ~ 0.9–10 MPa [38]). Regarding previous research on 3D printed scaffolds with the same scaffold material and internal structure as ours, Kim *et al*. [18] found an elastic modulus of ~82 MPa for lattice scaffolds of PCL/30% (wt.%) nHAp with a height of 2.40 mm (6 layers). Our lattice scaffolds of PCL/30% (wt.%) nHAp with a height of ~2.34 mm (6 layers), however, yielded an elastic modulus of ~100 MPa. Also, Zimmerling *et al*. [14] reported an elastic modulus of ~40 MPa for the lattice scaffolds of PCL/30% (wt.%) nHAp with the height of 4.00 mm (10 layers), which is not in agreement with that obtained (*E* ~82 MPa) in our 10-layer lattice scaffolds with the height of ~3.9 mm. These inconsistent findings between studies, despite the same layer number and material type, may result from differences in parameters such as porosity level. However, there is lack of information with regard to the porosity level in these studies [14,18].

Another interesting finding of our study pertains to the difference between the mechanical properties of lattice and staggered scaffolds (Fig 8) while they both showed three regions upon compression (Fig 9). The reduction in mechanical properties of staggered scaffolds with increasing height appeared to be more pronounced than lattice scaffolds (Fig 8). This could be attributed to the effect of the shifting strands between subsequent layers in staggered scaffolds and consequently less mechanically supportive structure upon compression with increasing layer number. Crossover points between layers are responsible for load transmission and in lattice scaffolds, there are higher number of crossover points compared to staggered scaffolds, however, crossover points are not shared in staggered scaffolds [39].

It is worth discussing the strengths and limitations of the present study. First, this study aimed to explain the conflicting reports in the literature regarding the mechanical properties of PCL/HAp scaffolds and to further explore the influence of scaffold design on the mechanical properties for BTE. This research, however, is limited by a small sample size (n = 3 per internal structure and layer number) and, accordingly, low statistical power, which might be, in part, the reason for the lack of observed differences between scaffold groups with different heights. Next, we only assessed the microstructure of a single scaffold per structure/layer group and further research is warranted with a larger sample, possibly also incorporating other 3D imaging techniques (such as micro-computed tomography) in order to achieve more precise printability values. We also acknowledge that we did not evaluate the relationships between degradation rate and mechanical properties of scaffolds. This subject should be considered for future research since a tissue scaffold will experience degradation under *in-vitro* and/or *in-vivo* experimental conditions and the mechanical properties can be affected, accordingly [40,41].

## 5 Conclusions

It is important to study the mechanical properties of bone scaffolds since they provide a supportive template for cellular function in a bone defect site. However, the relationships between the design parameters and mechanical properties of PCL/nHAp scaffolds, as a promising composite scaffold for BTE, have not been well elucidated. For clarification, this research investigated the relationships between mechanical properties and the height of PCL/30% (wt.) nHAp scaffolds designed and 3D printed with lattice and staggered internal structures. It was found that height was an influential factor on mechanical properties of PCL/30% (wt.%) nHAp scaffolds due to changes in printability parameters for each layer including penetration between layers and pore size. Staggered scaffolds showed lower mechanical properties than the lattice scaffolds with the same height and were more sensitive to the change of scaffold height. Taken together, lattice scaffolds demonstrated the advantages of more stable mechanical properties over staggered scaffolds. Also, scaffolds with lower height were more promising in terms of mechanical properties compared to scaffolds with greater height.

## Supporting information

S1 File (DOCX)
