# Correction: Investigation into relationships between design parameters and mechanical properties of 3D printed PCL/nHAp bone scaffolds

**DOI:** 10.1371/journal.pone.0334458

**Published:** 2025-10-13

**Authors:** Zahra Yazdanpanah, Nitin Kumar Sharma, Amanda Zimmerling, David M. L. Cooper, James D. Johnston, Xiongbiao Chen

The second author’s initials appear incorrectly in the citation. The correct citation is: Yazdanpanah Z, Sharma NK, Zimmerling A, Cooper DML, Johnston JD, Chen X (2023) Investigation into relationships between design parameters and mechanical properties of 3D printed PCL/nHAp bone scaffolds. PLoS ONE 18(7): e0288531. https://doi.org/10.1371/journal.pone.0288531.

The ORCID iD is missing for the sixth author. Please see the author’s respective ORCID iD here:

- Author Xiongbiao Chen’s ORCID iD is: 0000-0002-4716-549X (https://orcid.org/0000-0002-4716-549X).

In S1 File, the caption of S9 Table, “p<0.05” has been replaced by “p≤ <0.05”. Please view the correct S1 File below.

## Supporting information

S1 File Supplementary material. (PDF)
