# Supplementary material for: Investigation into relationships between design parameters and mechanical properties of 3D printed PCL/nHAp bone scaffolds
Source: PLoS One. 2023 Jul 14;18(7):e0288531. doi: 10.1371/journal.pone.0288531 (PMC10348542; doi:10.1371/journal.pone.0288531)
Supplement: S1 File — (DOCX) [file pone.0288531.s001.docx]

**Supporting Information**

S1 Table. Statistical comparisons between diameters of strands in different scaffold groups (NS pertains to not significant, * *p* ≤ 0.05).

| **Bonferroni's multiple comparisons test** | **Summary** | **Adjusted *p* value** |
| --- | --- | --- |
| 4-layer lattice vs. 4-layer staggered | NS | >0.9999 |
| 6-layer lattice vs. 6-layer staggered | NS | 0.1469 |
| 8-layer lattice vs. 8-layer staggered | NS | >0.9999 |
| 10-layer lattice vs. 10-layer staggered | NS | >0.9999 |
| 4-layer lattice vs. 6-layer lattice | NS | >0.9999 |
| 4-layer lattice vs. 8-layer lattice | NS | >0.9999 |
| 4-layer lattice vs. 10-layer lattice | NS | >0.9999 |
| 4-layer staggered vs. 6-layer staggered | * | 0.0109 |
| 4-layer staggered vs. 8-layer staggered | NS | >0.9999 |
| 4-layer staggered vs. 10-layer staggered | NS | 0.8477 |

S2 Table. Statistical comparisons of distances between strands in different scaffold groups (NS pertains to not significant).

| **Bonferroni's multiple comparisons test** | **Summary** | **Adjusted *p* value** |
| --- | --- | --- |
| 4-layer lattice vs. 6-layer lattice | NS | 0.3839 |
| 4-layer lattice vs. 8-layer lattice | NS | 0.3513 |
| 4-layer lattice vs. 10-layer lattice | NS | >0.9999 |
| 4-layer lattice vs. 4-layer staggered | NS | >0.9999 |
| 6-layer lattice vs. 6-layer staggered | NS | >0.9999 |
| 8-layer lattice vs. 8-layer staggered | NS | 0.7563 |
| 10-layer lattice vs. 10-layer staggered | NS | >0.9999 |
| 4-layer staggered vs. 6-layer staggered | NS | >0.9999 |
| 4-layer staggered vs. 8-layer staggered | NS | >0.9999 |
| 4-layer staggered vs. 10-layer staggered | NS | >0.9999 |

S3 Table. The amount of penetration between 3D printed layers in lattice scaffolds with different numbers of layers (Data are presented as M ± SD).

| ***Δ_0_*** | **10-layer scaffold** | **8-layer scaffold** | **6-layer scaffold** | **4-layer scaffold** |
| --- | --- | --- | --- | --- |
| Layer 1 – Layer 2 | 0.250 ± 0.017 mm | 0.254 ± 0.019 mm | 0.276 ± 0.004 mm | 0.259 ± 0.022 mm |
| Layer 3 – Layer 4 | 0.201 ± 0.012 mm | 0.229 ± 0.019 mm | 0.183 ± 0.008 mm | 0.259 ± 0.023 mm |
| Layer 5 – Layer 6 | 0.200 ± 0.006 mm | 0.225 ± 0.034 mm | 0.147 ± 0.019 mm | NA |
| Layer 7 – Layer 8 | 0.172 ± 0.011 mm | 0.199 ± 0.012 mm | NA | NA |
| Layer 9 – Layer 10 | 0.172 ± 0.025 mm | NA | NA | NA |

S4 Table. The amount of penetration between 3D printed layers in staggered scaffolds with different numbers of layers (Data are presented as M ± SD).

| ***Δ_0_*** | **10-layer scaffold** | **8-layer scaffold** | **6-layer scaffold** | **4-layer scaffold** |
| --- | --- | --- | --- | --- |
| Layer 1 – Layer 2 | 0.264 ± 0.013 | 0.250 ± 0.022 | 0.270 ± 0.006 | 0.250 ± 0.017 |
| Layer 3 – Layer 4 | 0.250 ± 0.008 | 0.219 ± 0.010 | 0.180 ± 0.017 | 0.210 ±0.013 |
| Layer 5 – Layer 6 | 0.222 ± 0.016 | 0.184 ± 0.010 | 0.135 ± 0.017 | NA |
| Layer 7 – Layer 8 | 0.215 ± 0.014 | 0.153 ± 0.013 | NA | NA |
| Layer 9 – Layer 10 | 0.137 ± 0.008 | NA | NA | NA |

S5 Table. Pore width values in lattice scaffolds with different numbers of layers (Data are presented as M ± SD).

| ***Px*** | **10-layer scaffold** | **8-layer scaffold** | **6-layer scaffold** | **4-layer scaffold** |
| --- | --- | --- | --- | --- |
| Layer 1 – Layer 2 | 0.366 ± 0.008 | 0.373 ± 0.020 | 0.303 ± 0.037 | 0.352 ± 0.018 |
| Layer 3 – Layer 4 | 0.411 ± 0.008 | 0.361 ± 0.020 | 0.329 ± 0.009 | 0.379 ± 0.013 |
| Layer 5 – Layer 6 | 0.392 ± 0.020 | 0.399 ± 0.030 | 0.466 ± 0.030 | NA |
| Layer 7 – Layer 8 | 0.438 ± 0.034 | 0.397 ± 0.040 | NA | NA |
| Layer 9 – Layer 10 | 0.346 ± 0.070 | NA | NA | NA |

S6 Table. Pore height values in lattice scaffolds with different numbers of layers (Data are presented as M ± SD).

| ***Pz*** | **10-layer scaffold** | **8-layer scaffold** | **6-layer scaffold** | **4-layer scaffold** |
| --- | --- | --- | --- | --- |
| Layer 1 – Layer 3 | 0.110 ± 0.002 | 0.079 ± 0.007 | 0.079 ± 0.007 | 0.085 ± 0.008 |
| Layer 3 – Layer 5 | 0.149 ± 0.012 | 0.134 ± 0.031 | 0.187 ± 0.006 | NA |
| Layer 5 – Layer 7 | 0.179 ± 0.023 | 0.143 ± 0.022 | NA | NA |
| Layer 7 – Layer 9 | 0.176 ± 0.013 | NA | NA | NA |

S7 Table. Pore width values in staggered scaffolds with different numbers of layers (Data are presented as M ± SD).

| ***Px*** | **10-layer scaffold** | **8-layer scaffold** | **6-layer scaffold** | **4-layer scaffold** |
| --- | --- | --- | --- | --- |
| Layer 1 – Layer 2 | 0.323 ± 0.010 | 0.415 ± 0.020 | 0.380 ± 0.023 | 0.332 ± 0.002 |
| Layer 3 – Layer 4 | 0.364 ± 0.004 | 0.416 ± 0.040 | 0.420 ± 0.009 | 0.375 ± 0.040 |
| Layer 5 – Layer 6 | 0.367 ± 0.020 | 0.400 ± 0.040 | 0.418 ± 0.012 | NA |
| Layer 7 – Layer 8 | 0.375 ± 0.010 | 0.315 ± 0.060 | NA | NA |
| Layer 9 – Layer 10 | 0.524 ± 0.080 | NA | NA | NA |

S8 Table. Pore height values in staggered scaffolds with different numbers of layers (Data are presented as M ± SD).

| ***Pz*** | **10-layer scaffold** | **8-layer scaffold** | **6-layer scaffold** | **4-layer scaffold** |
| --- | --- | --- | --- | --- |
| Layer 1 – Layer 3 | 0.089 ± 0.007 | 0.108 ± 0.004 | 0.176 ± 0.004 | 0.099 ± 0.006 |
| Layer 3 – Layer 5 | 0.113 ± 0.004 | 0.190 ± 0.006 | 0.169 ± 0.007 | NA |
| Layer 5 – Layer 7 | 0.113 ± 0.007 | 0.153 ± 0.003 | NA | NA |
| Layer 7 – Layer 9 | 0.130 ± 0.005 | NA | NA | NA |

S9 Table. Statistical comparisons between heights in different scaffold groups (NS pertains to not significant, * *p*≤ 0.05).

| **Bonferroni's multiple comparisons test** | **Summary** | **Adjusted *p* value** |
| --- | --- | --- |
| 4-layer lattice vs. 6-layer lattice scaffolds | * | <0.0001 |
| 4-layer lattice vs. 8-layer lattice scaffolds | * | <0.0001 |
| 4-layer lattice vs. 10-layer lattice scaffolds | * | <0.0001 |
| 4-layer staggered vs. 6-layer staggered scaffolds | * | <0.0001 |
| 4-layer staggered vs. 8-layer staggered scaffolds | * | <0.0001 |
| 4-layer staggered vs. 10-layer staggered scaffolds | * | <0.0001 |
| 4-layer lattice vs. 4-layer staggered scaffolds | NS | >0.9999 |
| 6-layer lattice vs. 6-layer staggered scaffolds | NS | >0.9999 |
| 8-layer lattice vs. 8-layer staggered scaffolds | NS | >0.9999 |
| 10-layer lattice vs. 10-layer staggered scaffolds | NS | >0.9999 |

S10 Table. Statistical comparisons between elastic modulus in lattice and staggered scaffolds (NS pertains to not significant, * *p* ≤ 0.05).

| **Bonferroni's multiple comparisons test** | **Summary** | **Adjusted *p* value** |
| --- | --- | --- |
| 4-layer lattice vs. 6-layer lattice scaffolds | NS | 0.2976 |
| 4-layer lattice vs. 8-layer lattice scaffolds | NS | 0.6956 |
| 4-layer lattice vs. 10-layer lattice scaffolds | * | 0.0139 |
| 4-layer staggered vs. 6-layer staggered scaffolds | NS | >0.9999 |
| 4-layer staggered vs. 8-layer staggered scaffolds | * | 0.0067 |
| 4-layer staggered vs. 10-layer staggered scaffolds | * | 0.0145 |

S11 Table. Statistical comparisons between yield strength in lattice and staggered scaffolds (NS pertains to not significant, * *p* ≤ 0.05).

| **Bonferroni's multiple comparisons test** | **Summary** | **Adjusted *p* value** |
| --- | --- | --- |
| 4-layer lattice vs. 6-layer lattice scaffolds | NS | >0.9999 |
| 4-layer lattice vs. 8-layer lattice scaffolds | NS | 0.2942 |
| 4-layer lattice vs. 10-layer lattice scaffolds | * | 0.0107 |
| 4-layer staggered vs. 6-layer staggered scaffolds | * | 0.0069 |
| 4-layer staggered vs. 8-layer staggered scaffolds | * | 0.0011 |
| 4-layer staggered vs. 10-layer staggered scaffolds | * | <0.0001 |

S12 Table. Statistical comparisons between elastic modulus in lattice and staggered scaffolds with the same layer number (NS pertains to not significant, * *p* ≤ 0.05).

| **Bonferroni's multiple comparisons test** | **Summary** | **Adjusted *p* value** |
| --- | --- | --- |
| 4-layer lattice vs. 4-layer staggered scaffolds | NS | 0.8751 |
| 6-layer lattice vs. 6-layer staggered scaffolds | NS | >0.9999 |
| 8-layer lattice vs. 8-layer staggered scaffolds | * | 0.0043 |
| 10-layer lattice vs. 10-layer staggered scaffolds | NS | 0.6838 |

S13 Table. Statistical comparisons between yield strength in lattice and staggered scaffolds with the same layer number (NS pertains to not significant, * *p* ≤ 0.05).

| **Bonferroni's multiple comparisons test** | **Summary** | **Adjusted *p* value** |
| --- | --- | --- |
| 4-layer lattice vs. 4-layer staggered scaffolds | NS | >0.9999 |
| 6-layer lattice vs. 6-layer staggered scaffolds | * | 0.0001 |
| 8-layer lattice vs. 8-layer staggered scaffolds | * | 0.0006 |
| 10-layer lattice vs. 10-layer staggered scaffolds | * | <0.0001 |
