# Supplementary material for: Correction: Investigation into relationships between design parameters and mechanical properties of 3D printed PCL/nHAp bone scaffolds
Source: PLoS One. 2025 Oct 13;20(10):e0334458. doi: 10.1371/journal.pone.0334458 (PMC12517493; doi:10.1371/journal.pone.0334458)
Supplement: S1 File — (PDF) [file pone.0334458.s001.pdf]

Supporting Information

S1 Table. Statistical comparisons between diameters of strands in different scaffold groups (NS pertains to not significant, * $p \leq 0.05$).

**Bonferroni's multiple comparisons test**	**Summary**	**Adjusted *p* value**
4-layer lattice vs. 4-layer staggered	NS	>0.9999
6-layer lattice vs. 6-layer staggered	NS	0.1469
8-layer lattice vs. 8-layer staggered	NS	>0.9999
10-layer lattice vs. 10-layer staggered	NS	>0.9999
4-layer lattice vs. 6-layer lattice	NS	>0.9999
4-layer lattice vs. 8-layer lattice	NS	>0.9999
4-layer lattice vs. 10-layer lattice	NS	>0.9999
4-layer staggered vs. 6-layer staggered	*	0.0109
4-layer staggered vs. 8-layer staggered	NS	>0.9999
4-layer staggered vs. 10-layer staggered	NS	0.8477

S2 Table. Statistical comparisons of distances between strands in different scaffold groups (NS pertains to not significant).

**Bonferroni's multiple comparisons test**	**Summary**	**Adjusted *p* value**
4-layer lattice vs. 6-layer lattice	NS	0.3839
4-layer lattice vs. 8-layer lattice	NS	0.3513
4-layer lattice vs. 10-layer lattice	NS	>0.9999
4-layer lattice vs. 4-layer staggered	NS	>0.9999
6-layer lattice vs. 6-layer staggered	NS	>0.9999
8-layer lattice vs. 8-layer staggered	NS	0.7563
10-layer lattice vs. 10-layer staggered	NS	>0.9999
4-layer staggered vs. 6-layer staggered	NS	>0.9999
4-layer staggered vs. 8-layer staggered	NS	>0.9999
4-layer staggered vs. 10-layer staggered	NS	>0.9999

S3 Table. The amount of penetration between 3D printed layers in lattice scaffolds with different numbers of layers (Data are presented as M ± SD).

Δ_0	**10-layer scaffold**	**8-layer scaffold**	**6-layer scaffold**	**4-layer scaffold**
Layer 1 – Layer 2	0.250 ± 0.017 mm	0.254 ± 0.019 mm	0.276 ± 0.004 mm	0.259 ± 0.022 mm
Layer 3 – Layer 4	0.201 ± 0.012 mm	0.229 ± 0.019 mm	0.183 ± 0.008 mm	0.259 ± 0.023 mm
Layer 5 – Layer 6	0.200 ± 0.006 mm	0.225 ± 0.034 mm	0.147 ± 0.019 mm	NA
Layer 7 – Layer 8	0.172 ± 0.011 mm	0.199 ± 0.012 mm	NA	NA
Layer 9 – Layer 10	0.172 ± 0.025 mm	NA	NA	NA

S4 Table. The amount of penetration between 3D printed layers in staggered scaffolds with different numbers of layers (Data are presented as M ± SD).

Δ_0	**10-layer scaffold**	**8-layer scaffold**	**6-layer scaffold**	**4-layer scaffold**
Layer 1 – Layer 2	0.264 ± 0.013	0.250 ± 0.022	0.270 ± 0.006	0.250 ± 0.017
Layer 3 – Layer 4	0.250 ± 0.008	0.219 ± 0.010	0.180 ± 0.017	0.210 ±0.013
Layer 5 – Layer 6	0.222 ± 0.016	0.184 ± 0.010	0.135 ± 0.017	NA
Layer 7 – Layer 8	0.215 ± 0.014	0.153 ± 0.013	NA	NA
Layer 9 – Layer 10	0.137 ± 0.008	NA	NA	NA

S5 Table. Pore width values in lattice scaffolds with different numbers of layers (Data are presented as M ± SD).

Px	**10-layer scaffold**	**8-layer scaffold**	**6-layer scaffold**	**4-layer scaffold**
Layer 1 – Layer 2	0.366 ± 0.008	0.373 ± 0.020	0.303 ± 0.037	0.352 ± 0.018
Layer 3 – Layer 4	0.411 ± 0.008	0.361 ± 0.020	0.329 ± 0.009	0.379 ± 0.013
Layer 5 – Layer 6	0.392 ± 0.020	0.399 ± 0.030	0.466 ± 0.030	NA
Layer 7 – Layer 8	0.438 ± 0.034	0.397 ± 0.040	NA	NA
Layer 9 – Layer 10	0.346 ± 0.070	NA	NA	NA

S6 Table. Pore height values in lattice scaffolds with different numbers of layers (Data are presented as M ± SD).

Pz	**10-layer scaffold**	**8-layer scaffold**	**6-layer scaffold**	**4-layer scaffold**
Layer 1 – Layer 3	0.110 ± 0.002	0.079 ± 0.007	0.079 ± 0.007	0.085 ± 0.008
Layer 3 – Layer 5	0.149 ± 0.012	0.134 ± 0.031	0.187 ± 0.006	NA
Layer 5 – Layer 7	0.179 ± 0.023	0.143 ± 0.022	NA	NA
Layer 7 – Layer 9	0.176 ± 0.013	NA	NA	NA

S7 Table. Pore width values in staggered scaffolds with different numbers of layers (Data are presented as M ± SD).

Px	**10-layer scaffold**	**8-layer scaffold**	**6-layer scaffold**	**4-layer scaffold**
Layer 1 – Layer 2	0.323 ± 0.010	0.415 ± 0.020	0.380 ± 0.023	0.332 ± 0.002
Layer 3 – Layer 4	0.364 ± 0.004	0.416 ± 0.040	0.420 ± 0.009	0.375 ± 0.040
Layer 5 – Layer 6	0.367 ± 0.020	0.400 ± 0.040	0.418 ± 0.012	NA
Layer 7 – Layer 8	0.375 ± 0.010	0.315 ± 0.060	NA	NA
Layer 9 – Layer 10	0.524 ± 0.080	NA	NA	NA

S8 Table. Pore height values in staggered scaffolds with different numbers of layers (Data are presented as M ± SD).

Pz	**10-layer scaffold**	**8-layer scaffold**	**6-layer scaffold**	**4-layer scaffold**
Layer 1 – Layer 3	0.089 ± 0.007	0.108 ± 0.004	0.176 ± 0.004	0.099 ± 0.006
Layer 3 – Layer 5	0.113 ± 0.004	0.190 ± 0.006	0.169 ± 0.007	NA
Layer 5 – Layer 7	0.113 ± 0.007	0.153 ± 0.003	NA	NA
Layer 7 – Layer 9	0.130 ± 0.005	NA	NA	NA

S9 Table. Statistical comparisons between heights in different scaffold groups (NS pertains to not significant, * $p \leq 0.05$).

**Bonferroni's multiple comparisons test**	**Summary**	**Adjusted *p* value**
4-layer lattice vs. 6-layer lattice scaffolds	*	<0.0001
4-layer lattice vs. 8-layer lattice scaffolds	*	<0.0001
4-layer lattice vs. 10-layer lattice scaffolds	*	<0.0001
4-layer staggered vs. 6-layer staggered scaffolds	*	<0.0001
4-layer staggered vs. 8-layer staggered scaffolds	*	<0.0001
4-layer staggered vs. 10-layer staggered scaffolds	*	<0.0001
4-layer lattice vs. 4-layer staggered scaffolds	NS	>0.9999
6-layer lattice vs. 6-layer staggered scaffolds	NS	>0.9999
8-layer lattice vs. 8-layer staggered scaffolds	NS	>0.9999
10-layer lattice vs. 10-layer staggered scaffolds	NS	>0.9999

S10 Table. Statistical comparisons between elastic modulus in lattice and staggered scaffolds (NS pertains to not significant, * $p \leq 0.05$).

**Bonferroni's multiple comparisons test**	**Summary**	**Adjusted *p* value**
4-layer lattice vs. 6-layer lattice scaffolds	NS	0.2976
4-layer lattice vs. 8-layer lattice scaffolds	NS	0.6956
4-layer lattice vs. 10-layer lattice scaffolds	*	0.0139
4-layer staggered vs. 6-layer staggered scaffolds	NS	>0.9999
4-layer staggered vs. 8-layer staggered scaffolds	*	0.0067
4-layer staggered vs. 10-layer staggered scaffolds	*	0.0145

S11 Table. Statistical comparisons between yield strength in lattice and staggered scaffolds (NS pertains to not significant, * $p \leq 0.05$).

**Bonferroni's multiple comparisons test**	**Summary**	**Adjusted *p* value**
4-layer lattice vs. 6-layer lattice scaffolds	NS	>0.9999
4-layer lattice vs. 8-layer lattice scaffolds	NS	0.2942
4-layer lattice vs. 10-layer lattice scaffolds	*	0.0107
4-layer staggered vs. 6-layer staggered scaffolds	*	0.0069
4-layer staggered vs. 8-layer staggered scaffolds	*	0.0011
4-layer staggered vs. 10-layer staggered scaffolds	*	<0.0001

S12 Table. Statistical comparisons between elastic modulus in lattice and staggered scaffolds with the same layer number (NS pertains to not significant, * $p \leq 0.05$).

**Bonferroni's multiple comparisons test**	**Summary**	**Adjusted *p* value**
4-layer lattice vs. 4-layer staggered scaffolds	NS	0.8751
6-layer lattice vs. 6-layer staggered scaffolds	NS	>0.9999
8-layer lattice vs. 8-layer staggered scaffolds	*	0.0043
10-layer lattice vs. 10-layer staggered scaffolds	NS	0.6838

S13 Table. Statistical comparisons between yield strength in lattice and staggered scaffolds with the same layer number (NS pertains to not significant, * $p \leq 0.05$).

**Bonferroni's multiple comparisons test**	**Summary**	**Adjusted *p* value**
4-layer lattice vs. 4-layer staggered scaffolds	NS	>0.9999
6-layer lattice vs. 6-layer staggered scaffolds	*	0.0001
8-layer lattice vs. 8-layer staggered scaffolds	*	0.0006
10-layer lattice vs. 10-layer staggered scaffolds	*	<0.0001
